# Response to Letter to the Editor from Christian Koeder 

**DOI:** 10.5414/ALX02573E 

**Published:** 2025-03-06

**Authors:** Imke Reese, Christiane Schäfer, Barbara Ballmer-Weber, Kirsten Beyer, Sabine Dölle-Bierke, Suzanne van Dullemen, Uta Jappe, Sabine Schnadt, Regina Treudler

**Affiliations:** 1 Nutrition Therapy, Munich,; 2 NutritionTherapy, Schwarzenbek, Germany,; 3 Clinic for Dermatology and Allergology, Cantonal Hospital St. Gallen, St. Gallen,; 4 Department of Dermatology, University Hospital Zürich, Switzerland,; 5 Clinic for Pediatrics with focus on Pneumology and Immunology, Charité-Universitätsmedizin – Campus, Virchow-Klinikum,; 6 Allergology and Immunology, Department of Dermatology, Venereology and Allergology, Charité-Universitätsmedizin Berlin, Berlin,; 7 University Clinic Frankfurt, Clinic for Pediatrics, Frankfurt/Main,; 8 Division of Clinical and Molecular Allergology, Research, Center Borstel, German Center for Lung Research (DZL) Airway Research Center North (ARCN), Borstel,; 9 Interdisciplinary Allergy Outpatient Clinic, Department of Pneumology, University of Lübeck, Lübeck,; 10 German Allergy and Asthma Association (DAAB), Mönchengladbach, and; 11 Department of Dermatology, Venereology and Allergology, Leipzig University Medical Center, Leipzig, Germany

## Abstract

None.

Dear Doctor Koeder, – Thank you for your interest in the position paper of the DGAKI working group food allergy on “Vegan Diets from an Allergy Point of View” [1]. We are happy to address your comments and questions as follows: 

Dairy alternatives: Fortified soy drinks are explicitly mentioned as an adequate milk alternative – not only in the position paper but also in the current guideline for the management of IgE-mediated food allergy [2]. “Therefore, when avoiding cow’s milk, allergy societies recommend preferably using a calcium-enriched soy product as a milk substitute in order to adequately meet protein and calcium requirements.” Please see also section 4.2 titled “soy-based milk substitutes – wrongly criticized”. 

Vitamin B12: Our aim was to underline that a supplementation with vitamin B12 is necessary. Thank you for your comment that it can also be given weekly. 

EPA/DHA: We are not aware of any study showing a relevant rise in the omega-3 index (O3I) after supplementation with α-linoleic-acid. In a scoping review analyzing randomized controlled trials from January 2010 to September 2020 Lane et al. [3] clearly showed that supplementation with plant-based omega-3 oils did not result in a rise of O3I. Some studies even showed reductions. 

Calcium: We agree that we do need more studies on bioavailability of calcium, especially from plant-based drinks. Only fortified soy-based drinks seem to be adequate [4, 5, 6]. A recent study on bioaccessibility of calcium from plant-based drinks showed dramatic negative results [7]. Moreover, numerous studies on bone mineral density (BMD) in people avoiding milk show a high risk of low BMD in every age group [8, 9, 10, 11, 12, 13, 14, 15, 16, 17]. These findings support the view that adequate calcium supply may be difficult. 

VeChi Diet Study: We would like to apologize for the error that occurred during translation of the German position paper into English: not vitamin B12 but vitamin B2 was only met to a limited extent by the vegan group of the VeChi Diet Study. This error has already been corrected [18]. 

Allergy risk and dietary diversity: The foods that were associated with protection against development of allergic diseases were mostly animal-based foods such as milk, yoghurt, fish, eggs [19, 20]. 

Nutrient profile of vegan diets: A diet, which consists entirely of plant-based foods, cannot supply all nutrients (in sufficient quantities) unless these are supplemented (either by nutritional supplements or by fortified foods). Only vegans with regular supplementation will have an adequate nutrient status. 

Nutrient knowledge required: Without awareness that a vegan diet by itself is not sufficient, there will be no supplementation. Moreover, implementation of good sources of critical nutrients is only possible, if there is enough knowledge to be able to choose these “good sources”. Therefore, only those who are well informed have a good basis for ensuring that their nutrient requirements are covered. 

V-label: We just point to the fact that labelled vegan foods may not be free from animal products as allergy sufferers might assume. Thank you for the information that the Plamil vegan logo does not allow unintended contamination. 

Vegan diets for infants: “Critical” does not mean impossible, but that a high awareness is required. However, every elimination diet should be guided by healthcare professionals/dieticians especially in sensitive phases such as growth. 

Thank you for your comments and questions which allowed us to clarify certain aspects of our position paper. 

## Funding 

No funding. 

## Conflict of interest 

R. Treudler, S. Schnadt, S. van Dullemen, B. Ballmer-Weber, U. Jappe declare no conflict of interest. 

I. Reese declares the following conflicts of interest outside the submitted work: Honoraria: AGPAS e. V., Aimmune Therapeutics Germany GmbH, AlbertZwei media GmbH, ALK-Abello Arzneimittel GmbH, Beiersdorf Dermo Medical GmbH, BVDD e. V., DAAB e.V., DAEM e.V., Danone Deutschland GmbH, DAPM e. V., DDG e.V., DGAKI, DWA, GPA e.V., GEKA mbH, GWT-TUD GmbH, Helmholtz-Zentrum München, InfectoPharm Arzneimittel und Consilium GmbH, Kneipp-Ärztebund e.V., Leo Pharma Gmbh, Medical Project Design, MVS Medizinverlage, Nestlé Deutschland AG, Netz für entzündliche Dermatosen in Hannover e.V., Novartis Pharma GmbH, RG Ärztefortbildung, Sanomega GmbH, Schweizer Milchproduzenten SMP, SWR, Uniklinikum Augsburg, Uniklinikum TU München, Uniklinikum Marburg, VDD e.V., VDOe e.V., Vfed e.V. Publishers: Dustri, de Gruyter, Springer, Thieme, Münchner Verlagsgruppe. Reimbursement of travel costs: DGAKI, EAACI, GA2LEN GAFA. Advisory board activity: DAAB, DGAKI. 

C. Schäfer declares the following conflicts of interest outside the submitted work: Travel costs, honoraria: abbvie Deutschland GmbH & Co. KG, Aimmune Therapeutics Germany GmbH, DAAB e. V., DAEM e. V., DGAKI e. V., DGEM e. V., Dustri Verlag, GU Verlag, MVS Medizinverlage, Nestle Deutschland AG, Novartis Pharma GmbH, Roxall Medizin GmbH, Sanofi-Aventis Deutschland GmbH, Springer Verlag, Thieme Verlag, VFED e. V. Advisory board activity: DAAB e. V., NNI, Quetheb e.V. 

K. Beyer declares the receipt of consultation fees and/ or honoraria by Danone/Nutricia, Hipp und Nestlé, as well as research grants by Danone/Nutricia and Hipp outside the submitted work. 

S. Dölle-Bierke declares the receipt of honoraria by ALK and DAAB e. V., as well as research grants by BMBF (01EA2107B) outside the submitted work. 
